# Reliability of remote self-administered digital cognitive assessments: preliminary validation study

**DOI:** 10.3389/fdgth.2025.1571053

**Published:** 2025-07-03

**Authors:** Duong Huynh, Siao Ye, Reza Hosseini Ghomi, Mary Patterson, Bin Huang

**Affiliations:** ^1^ Department of Clinical Development, BrainCheck Inc., Austin, TX, United States; ^2^ Department of Neurology, Faculty, Institute for Neuroengineering, & eScience Institute, University of Washington, Seattle, WA, United States

**Keywords:** dementia diagnosis, digital cognitive assessment, self-administered testing, BrainCheck, reliability

## Abstract

**Introduction:**

Early diagnosis of cognitive impairment and dementia relies on comprehensive, evidence-based cognitive assessments, which currently requires a clinic visit and access to skilled healthcare providers. This poses a challenge for people who live in areas with inadequate primary care services and those who have economic, insurance, or other transient hardships (transportation, time, etc.) that limit their access to healthcare services. Digital cognitive assessments (DCAs) with remote testing capabilities have emerged as an efficient and cost-effective solution. The aim of this study was to validate the reliability of BrainCheck, a platform for DCAs, when self-administered remotely.

**Methods:**

A total of 46 participants (60.9% female; age range 52–76) remotely completed a battery of six BrainCheck cognitive assessments twice on the same device (iPad = 8, iPhone = 5, laptop = 33): the participants self-administered in one session and were administered by a research coordinator (RC) in the other session. Thirty participants completed the self-administered session first, while 16 completed it second. The inter-session interval (ISI) varied across participants, from within the same day to 21 days apart. Testing outcomes, including the duration of time needed to complete the battery, the raw score from each assessment, and the raw overall score, were compared between the two sessions.

**Results:**

We found moderate or good agreement between self- and RC-administered performance, with intraclass correlation ranging from 0.59 to 0.83. Results from mixed-effects modeling further confirm the non-significant difference between self- vs. RC-administered testing performance, which is independent of other factors including testing order, ISI, device, and participants’ demographic characteristics.

**Discussion:**

These results demonstrate the feasibility of remote self-administration using BrainCheck.

## 1 Introduction

Dementia has become a public health concern worldwide with significant social and economic impacts (1–4). Early diagnosis of dementia is crucial for timely interventions, care planning, and reducing healthcare costs (5). However, early diagnosis relies on comprehensive, evidence-based cognitive assessments, which currently requires a clinic visit and access to trained healthcare providers. On the patient side, this poses a challenge for people who live in rural or underserved areas with inadequate medical services and those who have health, economic, insurance, or other transient hardships (transportation, time, etc.) that limit their access to healthcare services (6). On the provider side, with the shortage of specialists like geriatricians and neurologists (7, 8), primary care providers (PCPs) are often on the front lines of providing dementia care (9). However, many are constrained by short appointment times and limited staff (10), which makes it difficult to offer routine cognitive assessment to meet the growing needs of an aging population and the rising prevalence of the disease. These challenges highlight a critical need for a better solution to improve the accessibility of cognitive assessments in the primary care setting.

Remote cognitive assessment has emerged as a promising solution to improve accessibility, particularly in light of the limitations on in-person care imposed by the COVID-19 pandemic. With this shift, various remote cognitive testing methods have been developed. Videoconference-based teleneuropsychology facilitates real-time interaction between patients and providers, making it possible to administer conventional in-person cognitive tests remotely under supervision (11). However, many patients may lack access to the required equipment (e.g., videoconference capable devices) or struggle with technical complications during setup. Telephone-based testing is a convenient alternative (12), but may not be suitable for patients with hearing loss. Importantly, as assistance from a healthcare professional during testing is still required, these methods are not likely to be scalable and practical.

Recent development in innovative digital cognitive assessments (DCAs) that allow for self-administration presents a more efficient and cost-effective solution for remote testing by eliminating the need for professional oversight during testing. Unlike traditional paper-and-pen instruments, DCAs offer automated objective scoring and instant interpretation of results. Additionally, DCAs are able to capture granular measurements (e.g., response time) and provide multiple alternate sets of stimuli to minimize practice effects (13, 14). By allowing patients to complete assessments independently at home, self-administered DCAs can significantly improve the screening, diagnosis, and monitoring of dementia, alleviating the burden on healthcare providers and enhancing the efficiency of routine and repeated cognitive testing. Self-administered DCAs also offer psychometric advantages. The home environment may reduce test anxiety, leading to more accurate reflections of a patient's day-to-day cognitive abilities and enhancing ecological validity (15). These advantages position self-administered DCAs as a vital tool for addressing the growing demand for dementia care, ultimately contributing to improved patient outcomes and more efficient utilization of healthcare resources.

BrainCheck Assess (BC-Assess) is an evidenced-based DCA developed by BrainCheck (16–19). It consists of a brief battery of standardized assessments that evaluate multiple cognitive domains relevant to cognitive decline, such as memory, attention, and executive function. Previous validation studies have demonstrated that BC-Assess can reliably and sensitively measure cognitive decline among those with early cognitive impairment (16, 17). Designed for flexibility and accessibility, the full battery takes just 10–15 min to complete and can be administered by clinical support staff with minimal training in a clinical office setting or self-administered at home. Unlike other self-administered DCAs such as Cantab (20), BC-Assess is web-based, device-agnostic, and mobile-responsive, allowing users to take the test on any internet-connected device at any time without downloading an app or requiring technical setup. Other platforms, including TestMyBrain (21) and BRANCH (22–24), offer similar features, but BC-Assess is built specifically for clinical use. It offers direct integration with electronic health records (EHRs), includes clinical decision support in the results report, and provides a digital care planning tool for post-diagnosis support.

While BC-Assess supports remote, unsupervised testing, its effectiveness depends not only on the design of the assessments but also on the testing environment. When self-administered remotely, the lack of supervision and real-time support may make the testing vulnerable to variability in the testing environment, including issues with connectivity, device capability, and users' technical literacy (25). Within this context, this study aimed to evaluate the feasibility and reliability of BC-Assess on different types of devices by comparing individuals' performance in self-administered vs. examiner-assisted settings. Comparisons were performed in terms of the time taken to complete the assessments and their testing result in each assessment.

## 2 Materials and methods

### 2.1 Participants

Participants were recruited through convenience sampling, utilizing the personal networks of BrainCheck employees. Employees were encouraged to share study details within their networks, allowing interested individuals to volunteer. Additionally, participants were invited to refer other eligible individuals who might be interested, expanding the pool through a snowball recruitment approach. Interested participants received an invitation via email to complete a screening questionnaire. Eligible participants were those aged 50 years or older who self-reported as cognitively healthy, had no motor impairments that might affect their ability to complete cognitive assessments, and had no experience with BC-Assess prior to the study. Enrollment and data collection occurred between April 9, 2020, to May 4, 2020 during the COVID-19 pandemic. A total of 46 participants (60.9% female; age range 52–76, mean 64.0, standard deviation 5.8) participated in this study. Table 1 summarizes demographic characteristics of the participants.

**Table 1:** Demographics of the study sample.

Demographic characteristics	Self-administered first (*N* = 30)	RC-administered first (*N* = 16)	Total (*N* = 46)
Sex, *n* (%)			
Female	17 (56.7%)	11 (68.7%)	28 (60.9%)
Male	13 (43.3%)	5 (31.3%)	18 (39.1%)
Age, years			
Mean (SD)	64.1 (6.2)	63.9 (5.1)	64.0 (5.8)
Range	52–76	56–70	52–76
Education Level, *n* (%)			
>=12 years	19 (63.3%)	12 (75%)	31 (67.4%)
<12 years	1 (3.4%)	1 (6.3%)	2 (4.3%)
Not reported	10 (33.3%)	3 (18.7%)	13 (28.3%)
Device, *n* (%)			
iPad	5 (16.7%)	3 (18.7%)	8 (17.4%)
iPhone	1 (3.3%)	4 (25%)	5 (10.9%)
Laptop browser	24 (80%)	9 (56.3%)	33 (71.7%)
Inter-session interval (hours)			
Median (25–75th percentile)	62 (22–284)	19 (3–25)	27 (13–197)
Range	1–497	1–248	1–497

SD, standard deviation.

### 2.2 Procedure

Each participant completed BC-Assess remotely in two sessions: one where the participant self-administered the assessments and another where a research coordinator (RC) administered it. For the self-administered session, the participant received general instructions via email. During the RC-administered session, which took place amid the COVID-19 pandemic, the RC connected with the participant over the phone call or video chat. If requested, the RC provided assistance to help the participant get set up. The RC stayed on the phone or video throughout the test with the participant, answering any questions that arose. In both sessions, testing was taken on the same device for each participant, which could be either an iPad (*n* = 8), iPhone (*n* = 5), or laptop (*n* = 33), based on the participant's accessibility of devices and preference. The time interval between the two sessions varied across participants, from within the same day to 21 days apart. The order of the self-administered and RC-administered sessions was randomized across participants. Among the 46 participants, 30 completed the self-administered session first, while 16 completed it second (see Table 1).

### 2.3 Measurements

BC-Assess included six assessments measuring key cognitive abilities:

(1) Immediate Recognition: Participants were shown a list of 10 words and later asked to identify which of 20 words (10 original and 10 distractors) were presented earlier, assessing short-term memory through recognition.
(2) Trail Making A: Participants tapped numbered circles in sequential order as quickly as possible, assessing visual attention and processing speed.
(3) Trail Making B: An extension of Trail Making A, this task requires participants to alternate between tapping numbers and letters in order (e.g., 1-A-2-B), measuring cognitive flexibility and set-switching.
(4) Stroop: This assessment measures executive function and inhibitory control using a color-word interference paradigm. Participants were shown a target word in black and must find it in a 4  ×  3 grid where word colors varied: neutral (black), congruent (word and color match), or incongruent (word and color conflict).
(5) Digit Symbol Substitution: Participants were shown a key pairing symbols with digits and must quickly select the digit that corresponded to a target symbol, evaluating processing speed.
(6) Delayed Recognition: Similar to Immediate Recognition but with a time delay filled with assessments (2)-(5). Without seeing the original 10 words again, participants were asked which of 20 words (10 original and 10 distractors) were presented, testing the ability to recognize information after a delay.

A more detailed description of these assessments can be found in our previous studies (17, 18). At the beginning of each assessment, participants were presented with test instructions and engaged in an interactive practice session that included simulations of the actual test and feedback on any incorrect responses. This approach was designed to ensure participants understand the tasks well before the actual test. To minimize practice effects, BC-Assess includes built-in mechanisms for randomly generating alternative forms of each assessment. For example, in Trail Making A, the spatial positions of the numbered dots are randomized with each administration. Performance on each assessment was quantified by either accuracy- or reaction time-based measures (Table 2).

**Table 2:** Raw score (RS) metric and transformed score (TS) calculation for each assessment.

Assessment	Raw score metric	Transformed score
Immediate/delayed recognition	Number of correct responses^a^	TS = 100 × RS/MAX^c^
Stroop	Median reaction time^b^	TS = 100 × (1 − RS/MAX)
Digit symbol substitution	Number of correct responses per s^a^	TS = 100 × RS/MAX
Trail making A	Median reaction time^b^	TS = 100 × (1 − RS/MAX)
Trail making B	Median reaction time^b^	N/A

^a^ Higher score indicates better performance.

^b^ Lower score indicates better performance.

^c^ MAX represents the population maximum score of the assessment across all individuals in the BrainCheck normative database.

In addition to assessment-level scores, a battery-level overall score was calculated to provide a composite metric summarizing each participant's cognitive performance across multiple domains. The BC-Assess raw overall score was calculated as the mean of performance scores from all assessments in the BC-Assess (except the Trail Making B), where each assessment score had been transformed from its natural range into a common range [0,100], using the formula in Table 2. Although Trail Making B remains as an important component that informs clinicians' understanding and interpretation of an individual's cognitive functioning, it is excluded from the calculation of the overall score. This is because Trail Making B is administered only if Trail Making A is completed. This design is intended to reduce frustration for individuals with cognitive difficulties by avoiding the presentation of a significantly more difficult assessment following failure on a simpler one. Trail Making B places high demands on cognitive flexibility and set-switching, making it more challenging than Trail Making A. Therefore, including Trail Making B in the overall score would lead to missing data that is conditional on prior task performance. This could introduce bias and potentially skew the interpretation of overall cognitive functioning.

### 2.4 Data analysis

Participants' performance was evaluated through the following outcomes: the total duration of time needed to complete the battery, the raw score from each individual assessment in BC-Assess defined in Table 2, and the BC-Assess raw overall score. For each outcome, descriptive statistics were calculated separately for the self- and RC-administered sessions. The reliability of self-administered BC-Assess was examined by comparing participants' performance across the two testing sessions. Bland-Altman plots were used to identify systematic differences and evaluate the variability of the differences in performance between the two sessions. Paired-samples *t*-tests were used for significant testing of the mean of the differences. The consistency and absolute agreement of performance between the two sessions were measured by Pearson correlation (*r*) and intraclass correlation (ICC). To calculate ICC, a two-way mixed-effects model with administration mode (self- vs. RC-administered) as a fixed factor and participant as a random factor was used to determine between- and within-subject components of variance. ICC was then calculated as the ratio of between-subject variance to the total variance.

To further investigate the factors that might influence participants' performance, we applied two complementary modeling approaches:

*Approach-1. Absolute score modeling:* a mixed-effects linear regression model was run for each outcome to analyze the effects of administration mode and participant/session characteristics on performance. In the model, administration mode (RC-administered = 0; self-administered = 1) was included as the main fixed factor of interest. Five covariates were included as additional fixed factors:

(1) testing order (first test = 0; second test = 1): to capture any difference in the testing outcome due solely to learning effect;
(2) time point of test measured in hours (first test = 0): to capture the effect of inter-session interval on the second testing outcome;
(3) device type (with iPhone selected to be the baseline): to capture differences in the testing outcome between computer browser, iPad and the baseline;
(4) participant's age;
(5) participant's sex (female = 0; male = 1).

Interaction terms between administration mode and each of the five covariates were also included in the model to capture the possible influence of each factor on the difference in performance between the self- and RC-administered sessions. A random intercept by participant was included in the model to account for the effects of individual differences.

*Approach-2. Relative score difference modeling:* a fixed-effects linear regression model was run where each outcome variable was the within-subject difference in score between the self- and RC-administered sessions (self—RC) for each participant. This relative score difference modeling allowed us to examine whether the magnitude of discrepancy between administration modes could be systematically explained by participant or session-related variables. The predictors included delta testing order (+1 if self-administered testing occurred first; −1 otherwise), delta inter-session interval in hours (self-administered time minus RC-administered time), age, sex, and device type. Although participants used the same device in both sessions, device type was retained as a predictor in this model to assess whether the magnitude of performance difference varied across platforms. Age and sex were also included, as individual characteristics may relate to different patterns of performance change, even though they remained constant across sessions.

Together, the two approaches provide complementary insights. Approach 1 identifies which factors, including administration mode, are associated with absolute test performance across participants, accounting for individual differences. Approach 2 isolates the within-subject change between self- and RC-administered sessions, showing which factors explain variation in mode-related performance differences.

In both approaches, due to data missingness and the majority of participants having more than 12 years of education, education level was excluded from analysis. All non-categorical independent variables, as well as the dependent variable, were standardized to have a mean of 0 and standard deviation of 1 prior to model fitting. Model fitting was based on the Restricted Maximum Likelihood (REML) method for the mixed-effects model, and Ordinary Least Square (OLS) method for the fixed-effects model.

## 3 Results

The mean and standard deviation of each outcome are provided in Tables 3 for each administration mode.

**Table 3:** Mean (standard deviation), *t*-statistic from paired *t*-tests (degrees of freedom = 45), Pearson correlation *r*, intraclass correlation ICC, and their 95% confidence interval (CI) for each testing outcome.

Outcome	Self	RC	*t*-statistic	*r* (95% CI)	ICC (95% CI)
Duration (s)	835 (248)	801 (199)	1.16	0.62 (0.37–0.80)	0.59 (0.37–0.75)
BC-Assess overall score	78.8 (3.3)	79.9 (3.6)	−0.53	0.65 (0.39–0.79)	0.63 (0.42–0.78)
Immediate Recognition (correct responses)	18.7 (1.1)	18.8 (1.4)	−0.24	N/A	N/A
Delayed Recognition (correct responses)	18.3 (1.4)	18.3 (1.7)	0.0	N/A	N/A
Stroop (s)	2.31 (0.52)	2.22 (0.41)	2.43	0.85 (0.74–0.92)	0.83 (0.71–0.90)
Digit Symbol Substitution (correct responses per s)	0.39 (0.08)	0.39 (0.09)	−0.20	0.70 (0.52–0.84)	0.68 (0.49–0.81)
Trail Making A (s)	1.20 (0.33)	1.18 (0.38)	0.65	0.80 (0.63–0.90)	0.80 (0.66–0.88)
Trail Making B (s)	1.85 (0.61)	1.89 (0.59)	−0.51	0.74 (0.57–0.86)	0.73 (0.56–0.84)

Self, self-administered; RC, RC-administered.

**Figure 1:**
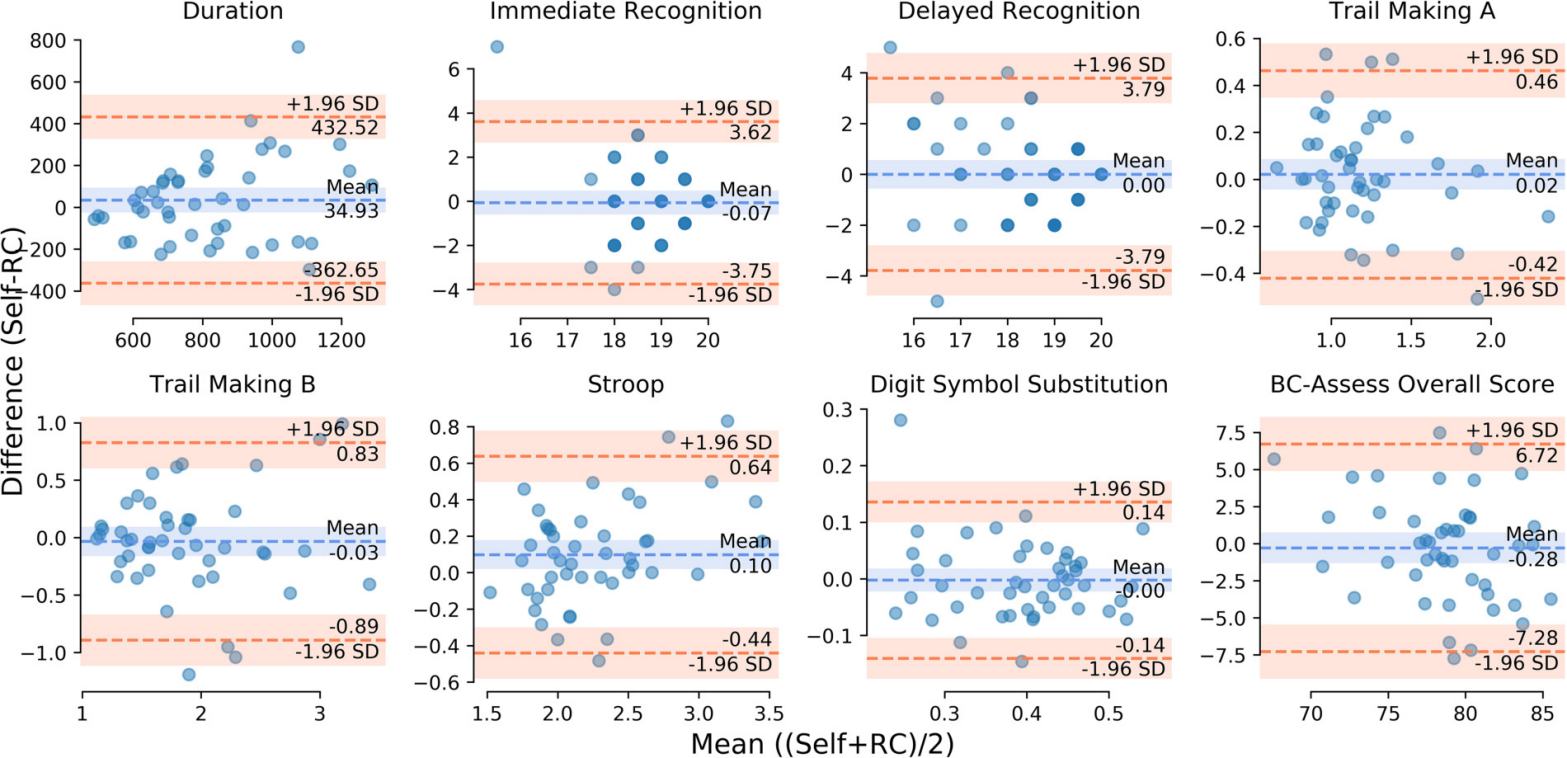
Bland-Altman plot for each testing outcome: differences between self- and RC-administered performance (*y*-axis) as a function of their means (*x*-axis). The orange dashed lines represent point estimates of the upper and lower limits of agreement (LoA: ±1.96 standard deviations of the differences). The blue dashed line represents point estimate of the mean of the differences. The shaded areas along the lines represent the corresponding 95% confidence intervals.

**Figure 2:**
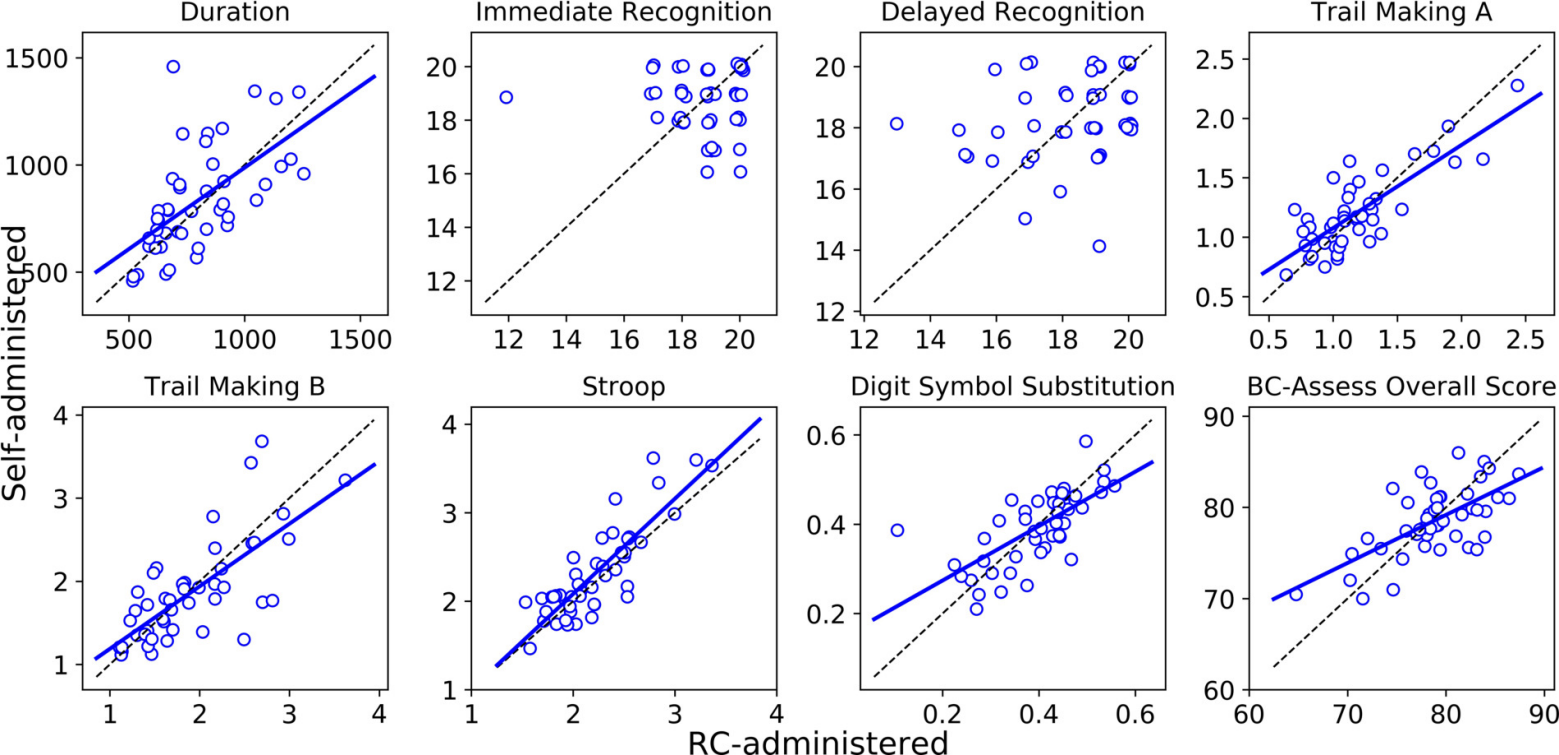
Linear regression (solid blue line) and the line of identity (dashed black line) for comparing self-administered performance (*y*-axis) against RC-administered performance (*x*-axis). Linear regression was not run for Immediate/Delayed Recognition due to the data showing no linear relationship.

**Figure 3:**
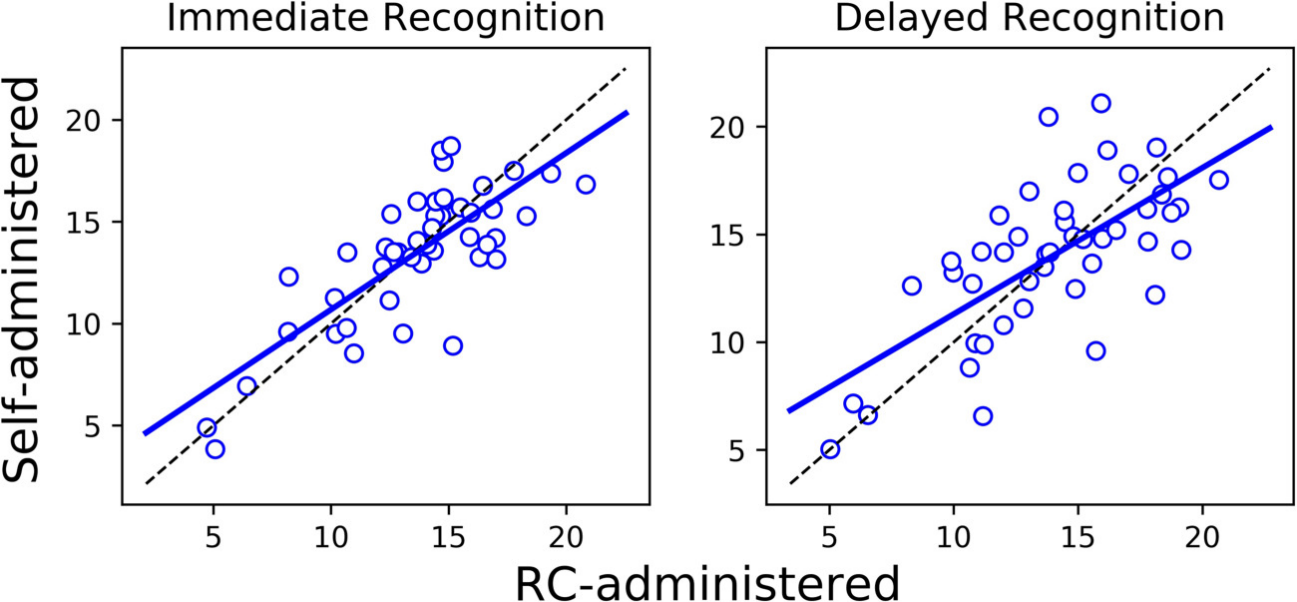
Linear regression (solid blue line) and the line of identity (dashed black line) for comparing self-administered performance (*y*-axis) against RC-administered performance (*x*-axis) for Immediate/Delayed Recognition, where performance is measured as the number of correct responses per median reaction time.

The Bland-Altman plots in Figure 1 show for each outcome the distribution of the differences in performance between the self- and RC-administered sessions as a function of their means. Results from paired-samples *t*-tests show non-significant differences between self- and RC-administered performance for all testing outcomes except for Stroop [*t*(45) = 2.43; *p* = 0.019]. We found moderate or strong consistency and agreement between self- and RC-administered performance for most outcomes, with Pearson correlation r in the range 0.62–0.85 and intra-class correlation ICC in the range 0.59–0.83 (Table 3 and Figure 2). Pearson correlation and ICC were not calculated for Immediate/Delayed Recognition because no linear relationship was observed for the outcome measures of these assessments. It is worth noting that, unlike the other assessments, performance in these assessments is based only on accuracy of responses and does not take into account reaction times. When taking reaction times into account (with performance measured as the number of correct responses per median reaction time), we observed moderate Pearson correlations of 0.79 (95% CI: 0.59–0.88) and 0.69 (0.49–0.82), and ICCs of 0.78 (95% CI: 0.64–0.87) and 0.69 (0.50–0.81), for Immediate and Delayed Recognition, respectively (Figure 3). For Stroop, although the difference between self- and RC-administered performance is significant, the difference mean of 0.1 s (Figure 1) is relatively small compared with the variance of Stroop raw scores across participants, reflected by a high ICC value for this assessment.

Standardized estimates of fixed effects obtained from the mixed-effects modeling (Approach-1) for each testing outcome data, along with their 95% confidence intervals, are provided in Table 4A. For all outcomes, we found that the main effect of administration mode and all interactions between administration mode and the other five factors were not significant. These results indicate that participants performed equally well when they self-administered and when they were administered the assessments by a professional, regardless of testing order, the time interval between the two tests, testing device, and participants' age and sex. The non-significant effect of administration mode is consistent with the results from the *t*-tests for all outcomes except the Stroop raw score where *t*-test result is significant.

**Table 4A:** Standardized estimates and their 95% confidence intervals for fixed effects obtained from mixed-effects modeling (Approach 1) of different testing outcomes.

Fixed effect	Outcome							
	Duration	IR	DR	TA	TB	ST	DS	OS
Administration mode (adm)	.48 [−1.05, 2.02]	.69 [−.79, 2.16]	.36 [−1.04, 1.77]	.09 [−.80, .97]	−.34 [−1.32, .64]	.34 [−.45, 1.12]	.34 [−.50, 1.18]	.48 [−.52, 1.48]
Testing order (ord)	−.11 [−.91, .69]	−.43 [−1.20, .34]	−.18 [−.99, .62]	−.18 [−.84, .48]	.07 [−.60, .75]	−.20 [−.79, .40]	**.** **66 [** **.** **08, 1** **.** **24]**	.13 [−.54, .79]
Inter-session interval (isi)	.02 [−.31, .35]	.30 [−.01, .61]	.04 [−.28, .36]	.00 [−.19, .19]	−.10 [−.33, .12]	−.03 [−.19, .14]	.07 [−.12, .25]	.17 [−.06, .39]
Device [browser] (dev1)	.07 [−1.02, 1.16]	.24 [−.81, 1.29]	.23 [−.88, 1.34]	**1** **.** **16 [** **.** **18, 2** **.** **14]**	−.76 [−1.74, .21]	.58 [−.30, 1.46]	−.59 [−1.43, .25]	−.50 [−1.45, .45]
Device [iPad] (dev2)	.10 [−1.13, 1.34]	.11 [−1.08, 1.30]	.34 [−.92, 1.60]	.05 [−1.07, 1.16]	−.85 [−1.96, .26]	−.38 [−1.38, .63]	−.06 [−1.01, .90]	.17 [−.91, 1.24]
Age (age)	.04 [−.3, .38]	.10 [−.23, .42]	−.19 [−.53, .15]	.29* [−.00, .58]	**.38 [.09**, .**68]**	**.39 [.13**, .**65]**	−.**51** [−.**76,** −.**26]**	−.**38** [−.**67,** −.**10]**
Sex [male] (male)	−.07 [−.78, .64]	.32 [−.37, 1.0]	.01 [−.71, .73]	−.28 [−.89, .34]	−.18 [−.79, .44]	−.06 [−.61, .49]	.35 [−.19, .87]	.32 [−.29, .92]
Adm:ord	−.33 [−1.45, .79]	.02 [−1.07, 1.10]	−.15 [−1.39, 1.09]	.26 [−.94, 1.46]	−.18 [−1.35, .99]	−.14 [−1.22, .94]	−.32 [−1.33, .69]	−.26 [−1.39, .87]
Adm:isi	0.20 [−.68, 1.08]	.20 [−.65, 1.05]	.38 [−.51, 1.28]	−.34 [−.86, .19]	−.05 [−.65, .55]	.14 [−.31, .59]	−.15 [−.66, .37]	.30 [−.33, .93]
Adm:dev1	.22 [−1.30, 1.73]	−.37 [−1.83, 1.08]	−.21 [−1.53, 1.11]	−.37 [−1.04, .30]	.37 [−.45, 1.19]	.03 [−.56, .61]	−.10 [−.80, .59]	−.15 [−1.01, .70]
Adm:dev2	−.22 [−1.95, 1.51]	.07 [−1.58, 1.73]	−.15 [−1.64, 1.35]	−.18 [−.94, .57]	.29 [−.63, 1.21]	−.35 [−1.01, .31]	.06 [−.72, .85]	.09 [−.88, 1.05]
Adm:age	−.11 [−.56, .35]	−.20 [−.64, .24]	.24 [−.16, .64]	−.08 [−.29, .12]	.05 [−.20, .30]	.03 [−.15, .21]	.01 [−.20, .23]	.04 [−.22, .30]
Adm:male	−.55 [−1.52, .43]	−.89 [−1.82, .04]	−.09 [−.94, .77]	.03 [−.41, .48]	−.08 [−.62, .46]	−.16 [−.55, .23]	.01 [−.45, .47]	−.29 [−.85, .27]

* Marginal *p*-value (*p* = .052).

Duration, total duration to complete the battery; IR, Immediate Recognition raw score; DR, Delayed Recognition raw score; TA, Trail Making a raw score; TB, Trail Making B raw score; ST, Stroop raw score; DS, Digit Symbol Substitution raw score; OS, BC-assess raw overall score. Significant effects (*p* < 0.05) are highlighted in bold.

Significant main effects of the covariates were found for certain testing outcomes. Testing order impacts only performance in Digit Symbol Substitution (*p* = 0.025), suggesting participants got better at encoding and matching the symbols or developed a better strategy to complete the task after the first test. Inter-session interval does not significantly impact performance on any assessment. The effect of device type is found for only Trail Making A. With iPhone as the reference, the significant difference in Trail Making A response time when using laptop browser (*p* = 0.020), and the non-significant difference when using iPad, reflect the advantages of searching and directly tapping visual items on touch-based devices (iPad and iPhone) compared with using a touchpad on laptops. The effect of age is significant for Trail Making B (*p* = 0.011), Stroop (*p* = 0.003), Digit Symbol Substitution (*p* < 0.001), and BC-Assess overall performance (*p* = 0.009).

Results from the relative score difference modeling (Approach 2) are presented in Table 4B. Testing order emerged as a significant predictor of performance differences for both Digit Symbol Substitution (*p* = 0.015) and Stroop (*p* = 0.001). In both cases, results from the linear regression model reflect that participants performed better during whichever session occurred second, regardless of whether it was self- or RC-administered, relative to the first, suggesting a learning or practice effect independent of administration mode. Notably, no significant effects of age, device type, or inter-session interval were found for any outcome in the difference score model. These results suggest that, beyond testing order effects for Digit Symbol Substitution and Stroop, other participant and session characteristics did not systematically influence the magnitude of performance differences between administration modes.

**Table 4B:** Standardized estimates and their 95% confidence intervals of fixed effects obtained from fixed-effects modeling (Approach 2) of different testing outcomes.

Fixed effect	Outcome							
	Duration	IR	DR	TA	TB	ST	DS	OS
Delta testing order	−.28 [−.72, .16]	−.30 [−.71, .11]	−.26 [−.70, .17]	−.18 [−.61, .26]	−.14 [−.58, .31]	**−**.**50** [−.**90, −.10]**	.**63 [.28**, .**98]**	.03 [−.38, .44]
Delta inter-session interval	.20 [−.23, .64]	.35 [−.05, .75]	.25 [−.18, .68]	−.04 [−.47, .39]	−.04 [−.48, .39]	.10 [−.29, .50]	.04 [−.30, .39]	.34 [−.07, .74]
Device [browser]	.08 [−1.04, 1.20]	−.27 [−1.32, .78]	−.24 [−1.35, .88]	−.61 [−1.72, .51]	.42 [−.72, 1.56]	−.01 [−1.04, 1.01]	−.11 [−1.0, .78]	−.18 [−1.23, .87]
Device [iPad]	−.17 [−1.13, 1.34]	.05 [−1.13, 1.23]	−.12 [−1.38, 1.13]	−.29 [−1.55, .97]	.39 [−.90, 1.67]	−.60 [−1.76, .55]	.08 [−.93, 1.09]	.10 [−1.08, 1.29]
Age (age)	−.13 [−.48, .22]	−.15 [−.48, .18]	.15 [−.20, .49]	−.15 [−.49, .20]	.02 [−.34, .37]	.02 [−.30, .33]	.03 [−.25, .31]	.05 [−.28, .37]
Sex [male]	−.28 [−.98, .42]	−.56 [−1.21, .09]	.09 [−.61, .78]	−.09 [−.78, .61]	−.05 [−.76, .65]	−.15 [−.79, .49]	−.05 [−.61, .50]	−.24 [−.89, .41]

Duration, Total duration to complete the battery; IR, Immediate Recognition raw score; DR, Delayed Recognition raw score; TA, Trail Making A raw score; TB, Trail Making B raw score; ST, Stroop raw score; DS, Digit Symbol Substitution raw score; OS, BC-assess raw overall score. Significant effects (*p* < 0.05) are highlighted in bold.

## 4 Discussion

In this study, we found that self-administration of BC-Assess resulted in testing performance comparable to that obtained through professional administration. Moderate to high consistency and agreement were observed across testing outcomes between the two administration methods, with Pearson correlations and ICCs ranging from 0.62 to 0.85 and 0.59 to 0.83, respectively. Findings from the mixed-effects model further reinforce this conclusion, showing no significant main effect of administration mode for any test outcome, and no significant interactions between administration mode and participant or session characteristics. This suggests that participants, regardless of age, sex, testing device, or the order and timing of sessions, performed similarly across self- and RC-administered assessments. These findings contribute to a growing body of research demonstrating the feasibility and reliability of unsupervised DCAs, highlighting key factors such as data quality and completion rates (24, 26), participant retention (27), participants' adherence and acceptability (28), test-retest reliability (24, 27, 29–32), and convergent validity with an in-person “gold standard” paper-based neuropsychological test (30–32). While previous studies have directly compared self- and examiner-administered assessments (24, 33), the current study offers a more comprehensive analysis by considering the effects of various session and participant characteristics, as well as including a broader range of assessments and cognitive domains.

We observed from the mixed-effects model a general negative association between age and performance across assessments that emphasized response time (see Table 2). Specifically, older age was associated with slower performance, reflected in positive standardized coefficients for Trail Making A (marginally), Trail Making B, and Stroop, where performance is evaluated solely based on response time. A similar trend was observed for Digit Symbol Substitution, where performance is measured as the number of correct responses per second; here, older age was associated with a negative standardized coefficient, indicating a decline in response efficiency with age. These associations contributed to a significant effect of age in the overall score, which integrates performance across assessments. The findings align with established evidence of age-related declines in processing speed, executive function, and attention (34–36). In contrast, the model showed no significant effect of age for Immediate and Delayed Recognition, which assess memory. While aging is known to affect memory (37, 38), the recognition tasks might not have been sufficiently demanding to capture such changes among healthy participants in this study. Interestingly, despite the significant effect of age on measures of response times, the model revealed no age-related differences in the total duration to complete the full battery. One possible explanation for this is that total duration covers not only the time spent on task execution but also differences in how participants navigate the testing process. For example, while some participants may take additional time during the practice sessions to fully understand the tasks, others may proceed through them more quickly.

We did not observe a clear linear relationship between scores from the self- and RC-administered sessions for Immediate and Delayed Recognition, and thus Pearson correlation and ICC were not calculated for these assessments due to violations of linearity and homoscedasticity assumptions. This lack of linearity likely reflects a ceiling effect and limited variability on the lower end of the score spectrum, due to the use of a healthy participant sample, rather than being an artifact of the self-administered format. Supporting this interpretation, results from our previous study (19) showed that, even when these assessments were administered in person by a professional, the distributions of test-retest differences in accuracy-based scores from Immediate and Delayed Recognition were similar to those observed in the current study. Ceiling effects have been noted in memory tests, including the verbal paired associates and word list tasks, from the Wechsler Memory Scales (WMS) (39), the Rey Auditory Verbal Learning Test (RAVLT) (40), and the California Verbal Learning Test (CVLT) (41), where performance variability is often limited among high-functioning individuals (42). These effects can obscure the ability to detect subtle individual differences and make it difficult to assess the true reliability of memory tasks in healthy populations, as the reduced score range complicates consistency evaluation. In this study, when response time was incorporated into the performance metric (i.e., number of correct responses per median reaction time), we observed strong correlations and ICCs for both assessments (Figure 3). Including reaction time introduces a continuous and more sensitive measure of individual differences, which increases variability across participants (43).

Practice effects were observed for the Digit Symbol Substitution assessment in both the absolute and the relative score difference approaches, and for the Stroop assessment only in the relative approach. These findings highlight the role of task familiarity and strategy adaptation, particularly in assessments involving processing speed and executive function. The discrepancy in the Stroop results suggests that the relative score approach may be more sensitive to subtle within-subject changes, as it isolates the effect of testing order by removing between-subject variability. In contrast, the mixed-effects model incorporates both within- and between-subject sources of variation, which may reduce sensitivity to smaller effects that are consistent at the individual level but not large enough to be detected across participants. Practice effects may explain why a significant Stroop difference between self- and RC-administered sessions was detected in the paired-sample *t*-test, particularly given the imbalance in administration order where 30 of the 46 participants completed the self-administered session first. As the *t*-tests included different components of variance, observed differences between self- and RC-administered sessions in this analysis may be influenced by a combination of factors, including practice effects. Conversely, although practice effects were significant in both models for the Digit Symbol Substitution, no significant difference emerged from the paired *t*-test for this assessment. Closer inspection of individual data showed that Stroop practice effects were pronounced for many participants, potentially driving the group-level results. In contrast, practice effects for Digit Symbol Substitution appeared more modest and consistent across individuals.

Although BrainCheck utilizes alternative test forms in all assessments to reduce content-specific practice effects, procedural learning, such as adapting to task structure, navigation, or timing, cannot be entirely eliminated. This is especially relevant for tasks like Digit Symbol Substitution (44), where participants may develop more efficient encoding strategies with repeated exposure, and Stroop (45, 46), where familiarity may enhance cognitive control and reduce interference. Practically, these findings highlight the importance of accounting for practice effects in repeated cognitive assessments. Without appropriate controls, familiarity with the task can lead to inflated improvements or obscure real cognitive declines. The fact that practice effects emerged across both self- and RC-administered sessions further supports the comparability of administration modes, suggesting that exposure, rather than supervision, drives these gains. Future studies with larger samples and repeated assessments across multiple time points are needed to better estimate the magnitude and timing of practice effects, and inform strategies to mitigate practice effects, such as optimizing test schedules and applying statistical corrections.

For the Trail Making A, performance differed across device types. Participants using touchscreen devices (iPad, iPhone) completed the task more quickly than those using laptops. This difference may be due to the task's requirement for rapid, sequential selection of spatially dispersed targets, where the touchscreen interface provides a more direct input method, in contrast to the slower, more mechanical navigation with a trackpad or mouse. Interestingly, no device effects were observed for other assessments such as Trail Making B, Digit Symbol Substitution, or Stroop. For Trail Making B, its greater cognitive demands might have reduced the relative influence of input mechanics on performance. The other assessments involve more centralized and repetitive inputs, where the type of input device may have less impact on performance, thus explaining the absence of a device effect. While the observed device-related variability is not a concern in the current within-subject design, where each participant used the same device in both sessions, it highlights the importance of applying device-specific corrections in studies involving direct comparisons between devices, especially for tasks that involve spatial or motor coordination. To support this, BrainCheck employs device-specific normative data to ensure valid performance evaluation and accurate comparisons across devices.

Performance in cognitive testing when self-administered remotely should not be immediately assumed to match that when administered in a provider office setting. Compared with in-person testing, remote and self-administered testing may introduce greater variability due to uncontrolled factors in the testing environment, such as unexpected interruptions, and the lack of in-time support. To ensure accuracy and validity, it is important to evaluate how well the assessments withstand such variability and how intuitive they are for users without assistance from a test administrator. Encouragingly, BC-Assess demonstrated to be highly reliable when self-administered remotely. The moderate to high consistency and agreement between self- and RC-administered testing sessions observed in this study are in similar ranges with previous evaluations of test-retest reliability, both in supervised settings for BrainCheck (18) and in unsupervised settings for other DCA tools such as Cantab and Neurotrack Cognitive Battery (29, 47, 48). Several design features of BC-Assess may contribute to its reliability in remote and self-administered testing, although these remain untested hypotheses and should be investigated further in future work. First, the adaptation of traditional paper-based tests into a digital format might help reduce errors that could arise in an unsupervised setting. For example, in the BrainCheck Trail Making A/B test, participants tap or click dots rather than drawing lines to connect them, as in the paper version. This digital format may help limit the variability introduced by more open-ended actions like freehand drawing, potentially making the task clearer and easier to perform accurately. Second, BC-Assess typically takes 10–15 min to complete. This shorter format may make it easier for individuals to find a quiet, uninterrupted time to finish the test. Lastly, the clear instructions and interactive practice session at the start of each test allow participants to practice as needed to fully understand the task before the actual test, helping reduce misunderstandings or mistakes during the actual test. Future studies that quantify usability, task comprehension, and within-subject variability could help determine whether and how these design features contribute to reliability in self-administered cognitive testing.

In this study, the participants were allowed to use their preferred devices to take the tests. The majority 71.7% of participants used laptops while only 10.9% and 17.4% used iPads and iPhones. At the time of data collection, BrainCheck DCAs on mobile devices were limited to iOS systems and required an app download. The low usage of iPads and iPhones could be due to the limited ownership of these iOS devices among the participants. Participants with limited digital skills or those who preferred not to download another app on their mobile device might end up choosing to use a laptop where BrainCheck could be run directly on a web browser. For optimal accessibility, it is essential that DCAs are compatible with a wide range of devices and simple in setup, allowing individuals of diverse backgrounds and levels of digital literacy to complete them with ease. Since the study, BC-Assess has been expanded and optimized to support any devices with a browser—whether a smartphone, tablet, or laptop—enabling users to start an assessment simply by clicking a link sent via email or text message, without the need to download an app. Based on the promising results from this pilot study and the recent improvements, our future studies will evaluate the usability and feasibility of BrainCheck DCAs for remote self-administered testing across diverse populations and devices in real-world settings.

While the results are promising, this study has several limitations that should be considered when interpreting the findings. First, the sample size in this pilot study is small, and inter-session intervals varied significantly between participants. Additionally, the study only examined two time points; a longitudinal study would be needed to provide further insights into the feasibility of using BC-Assess for long-term monitoring of cognitive health. Second, the study did not include participants with cognitive impairments, who may struggle to complete the assessments independently. This limits the generalizability of our findings to more vulnerable populations who might be the primary beneficiaries of cognitive assessments. Third, the study sample mainly consisted of participants with higher education levels, who are also likely to have higher digital literacy. Therefore, the results may not be applicable to individuals with lower education levels or limited digital literacy. Furthermore, the sample was drawn from a research setting rather than a real-world clinical environment, where contextual factors such as variability in support and test-taking conditions may influence feasibility and performance. Future studies with larger and more diverse samples are certainly needed to further assess the feasibility of self-administration in broader demographic settings.

Despite the limitations, this pilot study provides initial evidence demonstrating the feasibility and reliability of BC-Assess as a tool for remote cognitive evaluations. Effective use of remote and self-administered DCAs has the potential to improve the accessibility of cognitive care, particularly for rural and underserved populations, thereby addressing critical gaps in the current healthcare landscape.

## Data Availability

The datasets presented in this article are not readily available because the dataset used in this study is proprietary to BrainCheck Inc. and cannot be shared due to confidentiality agreements. As such, the data is not available for public distribution or by request to the corresponding author. However, interested parties may contact BrainCheck Inc. directly for inquiries related to access or collaboration opportunities involving the dataset. Requests to access the datasets should be directed to Bin Huang, bin@braincheck.com.
